# Economy in Nursing Department

**Published:** 1908-03-14

**Authors:** 


### Nursing Administration.

### ECONOMY IN THE NURSING DEPARTMENT.

The new edition of Burdett's "Hospitals and Charities"* is unusually strong in the section devoted to the nursing department. Year by year statistics are collected, classified and published relating to the size and cost of the nursing staff in hospitals all over the kingdom, and at length, after many years, certain conclusions begin to emerge from labours which at first seemed to result in merely a collection of scattered facts linked on no common basis of comparison. We have now before us the statistics for 1906, and the cost of the nursing department in some forty-six hospitals of importance in Great Britain is available for comparison. The number of hospitals which send in returns is far larger, but out of the ninety-four which have supplied figures relating to their nursing staff only these forty-six have made an attempt to estimate the cost of boarding their nurses, and without this key to the rate of expenditure per head it is impossible to form any just idea of the cost of the nursing department. The figures are extremely interesting. We append a list of the cost per head of the nurses at some typical hospitals with medical schools attached:—

| | |
|---|---|
| The London Hospital | £51 |
| St. Bartholomew's | 48 |
| Guy's | 32 |
| St. Thomas's | 54 |
| St. George's | 52 |
| The Middlesex | 47 |
| St. Mary's | 51 |
| University College | 41 |
| King's College | 50 |
| The Royal Free | 44 |
| Birmingham General Hospital | 38 |
| Sheffield Royal Infirmary | 44 |
| Oxford Radcliffe Infirmary | 35 |
| Edinburgh Royal Infirmary | 42 |
| Glasgow Western Infirmary | 37 |
| Belfast Royal Victoria | 38 |

The extreme difference is a matter of £19 per head as between the cost of a nurse at the Oxford Radcliffe Infirmary and the cost of a nurse at St. Thomas's Hospital, a difference of £14 per head between the cost of a nurse at the Birmingham General Hospital and St. George's Hospital. The cause of these differences is easily ascertainable by examining into the other figures supplied. The scale of salaries at St. Thomas's is remarkably higher than at the Radcliffe, the rate per bed in the one case being £9 7s. and in the other £4 6s. The character of the work and the high importance of the training school at St. Thomas's fully accounts for this. Moreover, St. Thomas's has been labouring under a shortage of the nursing staff until quite recently. Their nurses, comparatively few in number, have been well paid and highly efficient, and that this results in a low average of expenditure is shown by the fact that the average cost, of the nursing per bed at St. Thomas's is among the lowest for metropolitan general hospitals. A higher rate of salaries again contributes to account for the higher cost of the nurses at St. George's as compared with that at the Birmingham General Hospital. In the one case the rate of pay is £11 per bed, in the other case £5. It cannot be considered other than reasonable that the nursing staff in London hospitals of world renown should command a high rate of pay. The prestige of the hospital depends to a great extent on the excellence of the nursing, and on the quality of the teaching done in this connection, and although the work may be equally arduous in provincial hospitals and the responsibilities very heavy, they do not offer the same proportion of influential posts demanding administrative ability.

Housekeeping expenses contrast very favourably in London as compared with the country. Six of the big London hospitals show an average cost per head for nurses' board below £20. At Guy's the average is £12 2s., or just under 5s. a week. But, then, at Guy's they have a first-rate cook, and feast sumptuously for their money. Only two hospitals out of the forty-six which have furnished particulars spend as much as 10s. per week on the board of their nurses, and these are the Chester Infirmary and the Hospital for Women, Soho. It is now getting to be fully understood that any laxness in housekeeping tells immediately on the average cost of the nurse. If the higher cost of the individual nurse in the London hospitals with medical schools is due principally to the higher rates of salaries paid in these establishments, it is the question of board which tells in comparing one provincial hospital with another. The Leicester Infirmary, for instance, shows an average of £45 per head for each nurse, boarding them at the rate of £20 9s. a year, while the North Staffordshire Infirmary, with the same number of beds, shows an average cost per head of nearly nine pounds less, and boards its nurses at the rate of £17 per head. Such differences, minute though they may seem, work out at many hundreds a year in the case of large institutions.

We regret much to see no attempt made to grapple with the collective expenses of the Nurses' Home. The compilers of these useful statistics quote the expenditure on the Raphael Home at Guy's and estimate that at least ten pounds a head should be added on to the expense of each nurse for the accommodation she receives in the nursing home, accommodation which includes lodging, fuel, light, general upkeep, service, and often recreation. We believe that under these headings an enormous variation exists as regards economy. The nurses' home where everyone is uncomfortable is always an extremely costly institution, and to come to some clear understanding, by means of a separate system of accounts, as to the exact cost of keeping the home going, would often be to institute reforms of a much-needed character.

In one particular parsimony is still too often practised in both large and small hospitals. We allude to the rate of pay accorded to matrons. It is not reasonable to expect that women possessed of the high qualifications required to superintend a complex establishment can contentedly settle down for life on a salary of from £50 to £100 a year. The highest economy is to pay the superintendent well.

*"Burdett's Hospitals and Charities," price 7s. 6d., post free 7s. 11d.

